# Supplementary material for: The causal relationship between the human gut microbiota and pyogenic arthritis: a Mendelian randomization study
Source: Front Cell Infect Microbiol. 2024 Nov 26;14:1452480. doi: 10.3389/fcimb.2024.1452480 (PMC11629706; doi:10.3389/fcimb.2024.1452480)
Supplement: Supplementary file 1 [file DataSheet1.zip › mendelian test/ebi-a-GCST90027792.csv_leave_one_out.pdf]

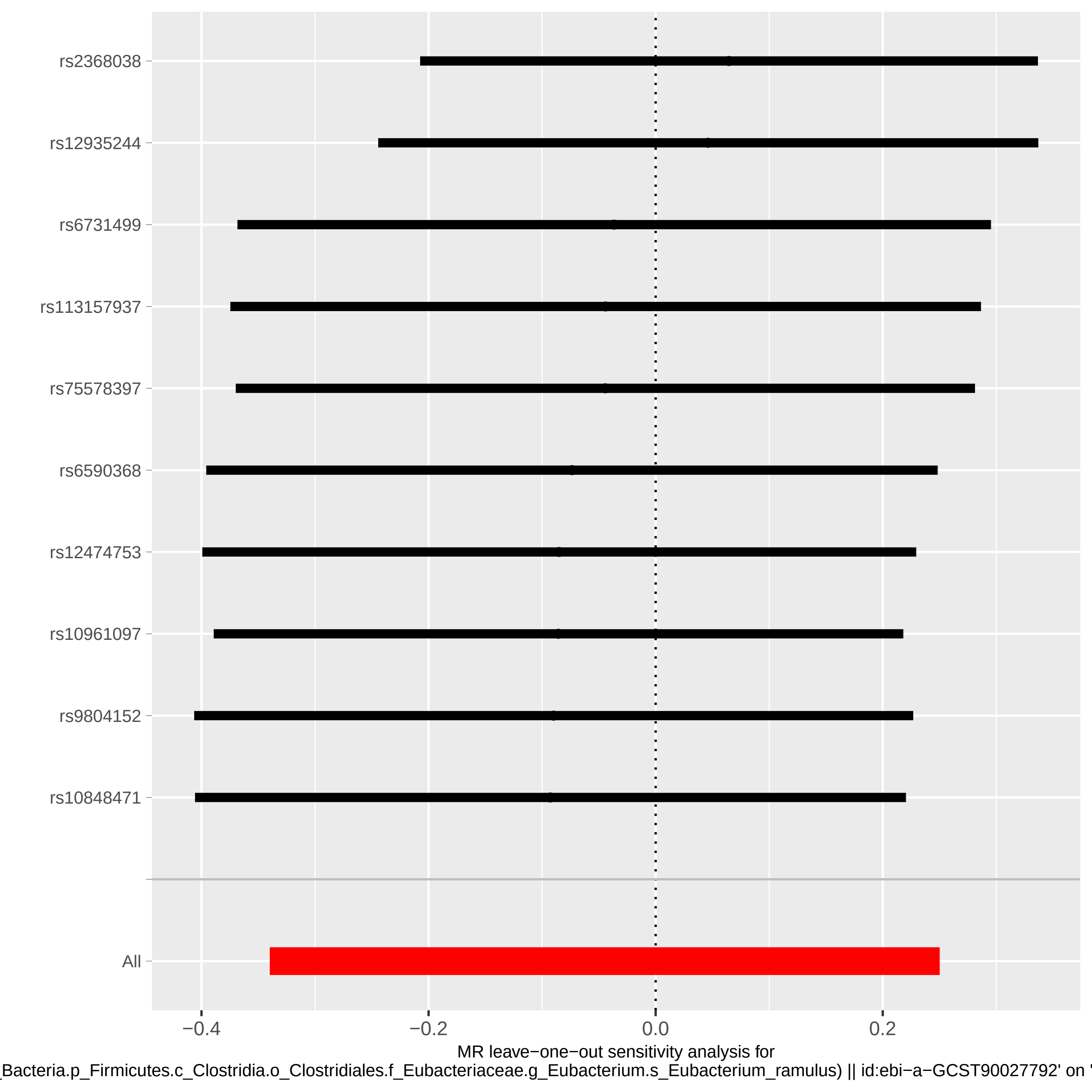

rs2368038
rs12935244
rs6731499
rs113157937
rs75578397
rs6590368
rs12474753
rs10961097
rs9804152
rs10848471
All
−0.4
−0.2
0.0
0.2
MR leave−one−out sensitivity analysis for
_Bacteria.p_Firmicutes.c_Clostridia.o_Clostridiales.f_Eubacteriaceae.g_Eubacterium.s_Eubacterium_ramulus) || id:ebi−a−GCST90027792' on '
